# Abdominal Surgery

**Published:** 1894-04-21

**Authors:** 


### Progress in Surgery.

### ABDOMINAL SURGERY.

Reed[1] thinks that the occurrence of suppuration during convalescence after cœliotomy and the subsequent development of ventral hernia are due to defective management of the abdominal incision. He lays stress on the desirability of going through the linea alba, and recommends closure of the wound by interrupted sutures of silkworm gut, passed from within outwards on both sides, entirely through the tissues. By this means tissues of similar nature are brought into apposition, and the possibility of infection is lessened. Schede[2] has used his buried wire sutures in 151 cœliotomies, in the majority of which there was risk of the subsequent development of ventral hernia. Small herniæ occurred in only seven cases, in which it was necessary to remove the wires prematurely. He first passes strong silver wire sutures through the entire thickness of the abdominal wall, but does not twist them. Then three or four silver sutures are introduced through the sheath of the rectus and the peritoneum only. The latter sutures are twisted, and then the deep sutures. The deep sutures are removed on the tenth day, and the patient allowed to go about without an abdominal bandage. Speaking of asepsis in abdominal surgery, Frank states[3] that no water to which any chemical germicide has been added should be brought in contact with the abdominal cavity, as results have shown that these strong germicides destroy the epithelium of the peritoneum and render septic absorption much easier. Moreover, they do not destroy the organisms; the best they can do is to retard their growth for a short time. In irrigation or sponging of the abdominal cavity it is, therefore, advisable to use sterilised water.

For this purpose filtration or distillation are not necessary, for sterilization can be effected by simply boiling the water and allowing it to cool. The most scrupulous cleanliness must be observed in the preparation of the patient, and the hands and instruments of the operator. Atmospheric infection is practically *nil*, for very few, if any, pyogenic microbes are found in air.

Pietro Lupo relates[4] a case of profuse bleeding from a stab in the epigastric region. Laparotomy was performed, and the hæmorrhage was found to come from two wounds on the inferior surface of the liver. Lupo stuffed each wound with a strip of aseptic gauze, the free end of which was left projecting from the abdominal wound. The patient died of septic peritonitis on the ninth day, the strips of gauze having been shortened but not removed. Lupo, while feeling encouraged by this case to arrest bleeding from a wounded liver by stuffing with gauze, says that in another case he will remove the strips on the third or fourth day, when there appears to be no further danger of renewed hæmorrhage, and will then close the abdominal wound, so as to minimise the risk of peritoneal infection.

Mangiagalli reports[5] two cases of tachycardia after cœliotomy. Both were cases of ovariotomy, the pulse ranging from 180 to 200 for several days after the operation, there being no elevation of temperature or evidence of sepsis. One patient died in collapse on the fifth day; the other recovered. The author thinks the phenomenon may have been due to the action of antiseptics upon the peritoneum, and hence upon the sympathetic ganglia. Garciadiego publishes[6] a method of diagnosing small effusions in the peritoneal cavity, which he believes to be accurate, and which is at the same time applicable even in those cases in which the existence of extreme tympanites would render percussion useless as a diagnostic measure. The method is as follows: Placing the patient on his back on an inclined plane of 45 deg., the index finger, well greased, is introduced into the rectum in order to examine the posterior peritoneal fold in men; in women, the posterior vaginal *cul-de-sac* is examined. If any effusion exists in the peritoneal cavity, no matter how small it may be, gravity will cause the liquid to descend to the most dependent parts, and in consequence of the position of the patient, it will gravitate towards the posterior part of the pelvic peritoneum. The exploring finger will clearly discover fluctuation, and to make sure that it is produced by a peritoneal effusion, it will suffice to change the patient's position, when it will be found that the fluctuation similarly varies according to the side to which the patient turns.

Mr. Greig Smith contributes[7] a paper on the so-called spontaneous disappearance of solid abdominal tumours. Three cases were recorded, the common leading features in which were the presence of a solid tumour in the abdomen, absence of pyrexia, clinical evidence of malignancy on abdominal section, and ultimate disappearance of the tumour, with complete restoration to health. In one case enterostomy was performed for the obstruction; in the other two, a fæcal fistula formed, closing spontaneously in the one and by operating in the other. Comparative observations were made on solid tumours of myomatous, tuberculous, and inflammatory nature, which, in the author's experience, had been found to disappear after operation. An explanation as to the origin of the tumours in question was made to rest on the process of phagocytosis and the heaping up of embryonic protective cells around a minute fistulous opening communicating with the intestine—possibly with a leaking Meckel's diverticulum. All the cases were movable tumours, and there was no question of cellulitic thickening. Cure was accidental, not spontaneous, depending in one case on a diversion of intestinal contents, and in the two others on an external opening of the fistula.

Llobet, after reporting[8] eight cases of hydatid cysts of the abdomen, with one death, remarks that the choice of procedure in the treatment of hydatids of the abdomen, and especially of the liver, is the immediate incision or method of Lindemann Landeau. In reality, the only other methods to be mentioned with it are that of puncture and double incision, or that of two stages. Both of these present inconveniences or dangers.

The first is inefficient in cases of multilocular cysts, and dangerous when the cysts are situated deeply. The second, or method of Volkmann, offers great security, but on the other hand does not allow exploration. The author thinks his results show that the method of immediate incision and drainage is not so dangerous as has been stated. The death reported occurred in a case of relapse followed by puncture.

[1] Therap. Gazette, Jan., 1894, and Amer. Journ., of Obstetrics, Sept., 1893. [2] Amer. Journ. of Med. Science, Nov., 1893, and Cent. für Gynäkologie, 1893, No. 31. [3] New York Med. Journ., Feb. 24, 1894. [4] Brit. Med. Journ., Dec. 30, 1893. [5] Amer. Journ. of Med. Science, Dec. 1893, p. 736. [6] Med. Record, Jan. 27, 1894. [7] Lancet and Brit. Med. Journ., Jan. 27, 1894. [8] Amer. Journ. of Med. Science, Mar. 1894, p. 336.

